# The renal and blood pressure response to low sodium diet in P2X4 receptor knockout mice

**DOI:** 10.14814/phy2.13899

**Published:** 2018-10-22

**Authors:** Eilidh Craigie, Robert I. Menzies, Casper K. Larsen, Grégory Jacquillet, Monique Carrel, Scott S. Wildman, Johannes Loffing, Jens Leipziger, David G. Shirley, Matthew A. Bailey, Robert J. Unwin

**Affiliations:** ^1^ Centre for Nephrology University College London Medical School London United Kingdom; ^2^ Institue for Anatomy University of Zürich Zürich Switzerland; ^3^ British Heart Foundation Centre for Cardiovascular Science University of Edinburgh Edinburgh United Kingdom; ^4^ Department of Biomedicine, Physiology Aarhus University Aarhus C Denmark; ^5^ Urinary System Physiology Unit Medway School of Pharmacy University of Kent Kent United Kingdom; ^6^ CVRM iMED AstraZeneca Gothenburg Gothenburg Sweden

**Keywords:** ENaC, hypertension, purinergic, purinoceptor, salt‐sensitivity, sodium

## Abstract

In the kidney, purinergic (P2) receptor‐mediated ATP signaling has been shown to be an important local regulator of epithelial sodium transport. Appropriate sodium regulation is crucial for blood pressure (BP) control and disturbances in sodium balance can lead to hypo‐ or hypertension. Links have already been established between P2 receptor signaling and the development of hypertension, attributed mainly to vascular and/or inflammatory effects. A transgenic mouse model with deletion of the P2X4 receptor (P2X4^−/−^) is known to have hypertension, which is thought to reflect endothelial dysfunction and impaired nitric oxide (NO) release. However, renal function in this model has not been characterized; moreover, studies in vitro have shown that the P2X4 receptor can regulate renal epithelial Na^+^ channel (ENaC) activity. Therefore, in the present study we investigated renal function and sodium handling in P2X4^−/−^ mice, focusing on ENaC‐mediated Na^+^ reabsorption. We confirmed an elevated BP in P2X4^−/−^ mice compared with wild‐type mice, but found that ENaC‐mediated Na^+^ reabsorption is no different from wild‐type and does not contribute to the raised BP observed in the knockout. However, when P2X4^−/−^ mice were placed on a low sodium diet, BP normalized. Plasma aldosterone concentration tended to increase according to sodium restriction status in both genotypes; in contrast to wild‐types, P2X4^−/−^ mice did not show an increase in functional ENaC activity. Thus, although the increased BP in P2X4^−/−^ mice has been attributed to endothelial dysfunction and impaired NO release, there is also a sodium‐sensitive component.

## Introduction

Extracellular ATP is an important signaling molecule acting through members of the purinergic P2 receptor family. P2 receptors are divided into two subgroups: P2X are ATP‐gated nonselective cation channels; P2Y are G protein‐coupled receptors. P2 receptors are widely expressed and, in the kidney, P2 receptor activation influences blood flow, glomerular filtration, and electrolyte reabsorption by the renal tubule (Burnstock et al. 2014). Indeed, abnormalities in the P2 system contribute to a range of renal diseases (Menzies et al. 2017a), including diabetic nephropathy (Menzies et al. 2017b). Normal renal function is essential for long‐term blood pressure (BP) regulation (Ivy and Bailey 2014), thus the P2 system is also a focus of hypertension research (Franco et al. 2015; Menzies et al. 2015a).

Efforts to resolve the mechanisms through which abnormal purinergic signaling causes hypertension have been supported by two developments. First, the development of receptor‐specific agonists, and particularly antagonists, has identified specific receptors contributing to experimental hypertension. For example, antagonism of P2X7 reduces BP in angiotensin II‐dependent hypertension by restoring the pressure natriuresis response (Menzies et al. 2013), promoting renal vasodilation (Franco et al. 2017) and increasing renal perfusion(Menzies et al. 2015b); a reduction in renal inflammation may also contribute to the long‐term antihypertensive effects of P2X7 antagonism (Menzies et al. 2017b).

Second, detailed physiological analysis of P2 knockout mice has provided a molecular understanding of the connection between specific receptors and BP regulation. For example, P2Y2 receptor knockout mice are hypertensive due in part to a loss of tonic inhibition of the epithelial Na^+^ channel (ENaC) (Pochynyuk et al. 2008, 2010; Rieg et al. 2008), which normally fine‐tunes urinary Na^+^ excretion to achieve sodium balance.

The current study takes the second of these approaches, using a global knockout mouse model to understand how loss of P2X4 function contributes to hypertension, as reported in human genetic association studies (Palomino‐Doza et al. 2008) (Stokes et al. 2011). P2X4 is the most abundant P2 receptor in the vascular endothelium (Wang et al. 2002) and activation by ATP underpins the normal vascular vasodilatory response to shear stress (Yamamoto et al. 2003). P2X4^−/−^ mice are hypertensive and have an impaired endothelial production of nitric oxide in response to increased vascular flow (Yamamoto et al. 2006). Such endothelial dysfunction would impair BP regulation through the pressure natriuresis response (Ivy and Bailey 2014), but P2X4 is also expressed in glomerular podocytes(Forst et al. 2016) and throughout the renal tubule (Turner et al. 2003). Whether loss of renal tubular P2X4 contributes to hypertension in P2X4^−/−^ mice is not known. P2X4 activation can influence ENaC activity in cell models (Wildman et al. 2005; Zhang et al. 2007; Thai et al. 2014) and in *ex vivo* in rat micro dissected CDs, activation of the receptor inhibits ENaC if ambient sodium concentration is high (Wildman et al. 2008). Further, ATP‐mediated inhibition of Na^+^ reabsorption in the thick limb of Henle is impaired in P2X4^−/−^ mice (Marques et al. 2012). Proposing that P2X4 activation normally exerts a tonic inhibition on sodium reabsorption in the distal nephron, we hypothesized that hypertension in P2X4^−/−^ mice would be alleviated by dietary sodium restriction. In the current study, we tested this hypothesis in vivo by assessing the effect of low sodium diet on BP and by measuring the net natriuretic response to distal tubule diuretics in P2X4^−/−^ mice and wild‐type controls.

## Methods and Materials

### Animals

All experiments were performed in accordance with the UK Home Office (Scientific Procedures) Act 1986 and Danish legislation on the protection of animals. P2X4 null mice (P2X4^−/−^) were a gift from GlaxoSmithKline. The generation of these mice has been described (Sim et al. 2006); they were bred to congenicity (>15 generations on C57BL6/J) and were maintained on a C57BL/6J background. A heterozygous‐cross breeding program was used and experimental wild‐type littermate controls were used throughout. Experiments were performed on mice aged 12–16 weeks. Mice were fed commercial rodent chow containing either 0.25% Na^+^ or 0.03% Na^+^ (Special Diet Services, Essex, UK).

### Renal clearance, blood pressure, and renal vascular resistance

Renal function experiments were performed as described previously (Craigie et al. 2012) in mice on a standard diet or after feeding a low Na^+^ diet for 14 days. Briefly, mice were anesthetized with thiobutabarbital Na^+^ (Inactin; 100 mg/kg IP) and intravenously infused with a solution containing 120 mmol/L NaCl, 15 mmol/L NaHCO_3_ and 5 mmol/l KCl. FITC‐labeled inulin (0.5% w:v) and p‐aminohippurate acid (PAH; 2% w:v) were added for measurement of glomerular filtration rate (GFR) and effective renal plasma flow (RPF), respectively. A 60‐min equilibration period was followed by two 40‐min periods of renal function measurements: period 1 (control collection) was followed by an IV bolus administration of either benzamil (2 mg/kg), bumetanide (1 mg/kg) or hydrochlorothiazide (HCTZ) (2 mg/kg) and after a 10‐min equilibration period the second collection period was initiated (Hunter et al. 2014). Mean arterial BP (MABP) was recorded continuously throughout via a carotid artery cannula (Powerlab, ADI, UK). At the end of each experiment, mice were euthanized by an intravenous dose of Inactin.

### Plasma and urine analyses

Sodium and potassium concentrations were measured by ion‐selective electrodes (Diamond SmartLyte, Diamond Diagnostics GmbH, Munich, Germany). Plasma and urine osmolality was measured using freezing‐point depression (Osmomart 030, Gonotec, Berlin, Germany). Blood volume was determined by IV injection of Evans Blue (1 *μ*L/g of a 0.5% wt:vol solution) in combination with hematocrit measurements. Urinary total nitric oxide (NOx) concentration was determined using a colorimetric enzyme assay (Enzo Life Sciences, Exeter, UK). Plasma samples for aldosterone measurements were collected directly from the vena cava of mice anesthetized with ketamine/xylazine combination; aldosterone concentration was measured by an aldosterone ELISA kit (DRG Diagnostics, Marburg, Germany).

### Calculations

The concentration of FITC‐Inulin in plasma and urine was determined by fluorimetric assay and those of PAH by colorimetric assay. The renal clearance of inulin and PAH was calculated in ml/min and used as an estimate of GFR and RPF, respectively. Renal blood flow (RBF) was then calculated in ml/min using the equation: RBF = RPF/1‐hematocrit value; renal vascular resistance (RVR) was calculated as MABP/RBF. Fractional excretion of electrolytes was calculated using the equation FE_*X*_ = E_*X*_/((P_*X*_ × GFR) × 100), where F_*X*_ = fractional X excretion, E_*X*_ = urinary X excretion, and P_*X*_ = Plasma X concentration. The net change in FENa following injection of the diuretic agents was calculated (ΔFENa) and used as an index of in vivo activity of ENaC (ΔFENa benzamil), NCC (ΔFENa thiazide), and NKCC2 (ΔFENa bumetanide).

### Renal tissue immunoblotting

Kidneys were lysed and total protein concentration was measured using the Bradford reagent (Bio‐Rad, California, USA) method. Membranes were incubated with primary antibodies detecting *α*‐, *β*‐, and *γ*‐ENaC, NCC (Sorensen et al. 2013), and NKCC2 (Wagner et al. 2008), followed by infrared dye‐conjugated secondary antibodies against rabbit and mouse IgGs (LI‐COR Biosciences, Bad Homburg, Germany), respectively. Separate gels were incubated with Proto Blue Safe Coomassie stain (National Diagnostics, North Carolina, USA) and analyzed by densitometry. These results were then used in the analysis of signals for normalization; analysis was performed with an infrared‐based imaging system (LI‐COR Odyssey, Nebraska, USA).

### Statistical analysis

Data are presented as mean ± SEM or dot plots with mean values. Experimental groups were compared by Student's *t*‐test or by ANOVA with appropriate *post‐hoc* testing in GraphPad Prism v.6.0 (San Diego, CA, USA).

## Results

### Dietary Na^+^ restriction normalizes blood pressure in P2X4^−/−^ mice

**Figure 1:**
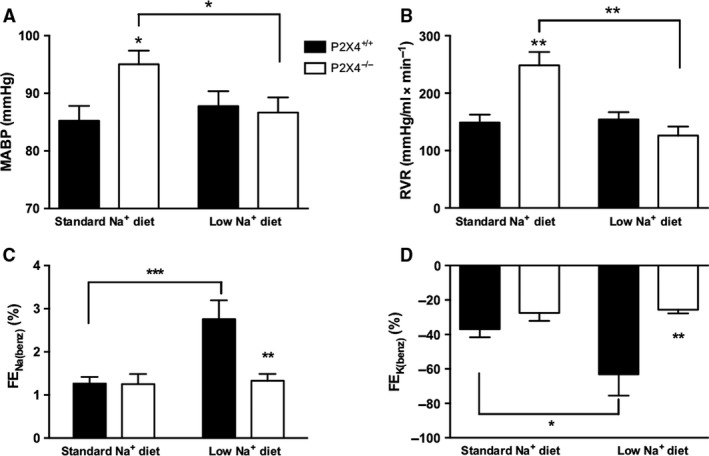
(A) Mean arterial blood pressure (MABP), (B) renal vascular resistance (RVR), (C) the absolute effect of benzamil on fractional excretion of Na^+^ (Δ FE_N__a(benz)_), and (D) K^+^ (Δ FE_K__(benz)_) in P2X4^+/+^ (■) and P2X4^−/−^ (□) mice on either a standard (*n* = 8 and 7, respectively) or a low Na^+^ diet for 14 days (*n* = 7 and 6, respectively). Data are mean ± SEM, and comparisons were made with ANOVA using Bonferroni *post‐hoc* testing. *P < 0.05; **P < 0.01; ***P < 0.001.

P2X4^−/−^ mice had elevated mean BP on a standard diet, approximately 10 mmHg higher than wild type (Fig. 1A). P2X4^−/−^ mice also had a significantly higher RVR (Fig. 1B). GFR was not different between genotypes (Table 1).

**Table 1:** Body weight and plasma parameters of P2X4^+/+^ and P2X4^−/−^ mice fed either a standard or a low Na^+^ diet for 14 days (*n* = 6 for each group)

	Standard sodium diet		Low sodium diet	
	P2X4^+/+^	P2X4^−/−^	P2X4^+/+^	P2X4^−/−^
Body weight (g)	25.8 ± 0.8	22.9 ± 1.7	25.8 ± 0.8	22.1 ± 2.5
Kidney weight (mg)	147 ± 3	173 ± 4	141 ± 10	174 ± 8
Plasma Na^+^ (mmol/L)	153.2 ± 0.5	152.2 ± 1.2	152.1 ± 1.1	154.2 ± 1.2
Plasma K^+^ (mmol/L)	4.4 ± 0.2	4.7 ± 0.2	4.2 ± 0.2	3.8 ± 0.1††
Blood volume (*μ*L/g BW)	106.6 ± 5.9	113. 2 ± 10.4	96.11 ± 8.0	76.61 ± 6.1*†
Plasma osmolality (mOsm/L)	310.8 ± 3.4	302 ± 5.2	312.5 ± 0.9	334.5 ± 5.7*††
Hematocrit (%)	44.7 ± 0.5	44.2 ± 1.1	44 ± 1.0	44.6 ± 0.8
GFR (mL/min/g kidney weight)	1.45 ± 0.14	1.73 ± 0.15	1.66 ± 0.12	1.40 ± 0.14

Data are mean ± SEM, and comparisons were made with ANOVA using Bonferroni *post‐hoc* testing. *P < 0.05, **P < 0.01 compared with P2X4+/+ mice fed the same diet; ^†^ *P* < 0.05, ^††^ *P* < 0.01 compared with same genotype fed a standard Na+ diet.

BP and renal hemodynamic measurements performed in cohorts of P2X4^−/−^ and wild‐type mice after 14 days on a low Na^+^ diet. The low Na^+^ diet did not affect BP in wild‐type mice but normalized BP of P2X4^−/−^ mice (Fig. 1A), reducing also RVR in these animals (Fig. 1B). GFR was not significantly affected by the low Na^+^ diet in either group (Table 1). We assessed blood volume in these cohorts of mice: low sodium diet significantly decreased blood volume compared with wild‐type mice (Table 1).

### Abnormal regulation of ENaC in P2X4^−/−^ mice

In light of the BP results and previous work showing that P2X4 activation can regulate ENaC function, we investigated whether functional ENaC activity was altered in P2X4^−/−^ mice. FENa (0.60 ± 0.12 vs. 0.82 ± 0.15%; *P* = 0.185) and FEK were similar between the genotypes on a standard Na^+^ diet. The ENaC blocker benzamil was then acutely administered to assess the contribution of ENaC transport to FE_Na_ and FE_K_. As expected, inhibition of ENaC caused a significant natriuresis and antikaliuresis (Fig. 1C). Our data suggest that ENaC reabsorbs ~1% of the filtered sodium load in both genotypes under standard conditions.

**Figure 2:**
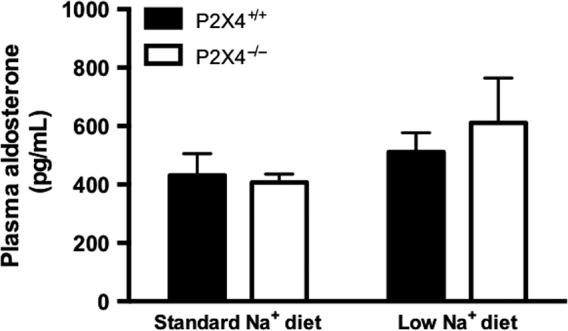
Plasma aldosterone concentration of P2X4^+/+^ (■) and P2X4^−/−^ (□) mice fed either a standard or a low Na^+^ diet for 14 days (*n* = 9 and 7, 8 and 7, respectively). Data are mean ± SEM, and comparisons were made with ANOVA using Bonferroni *post‐hoc* testing.

These experiments were then repeated in mice fed a low Na^+^ diet for 14 days to stimulate ENaC activity. In wild‐type mice, dietary sodium restriction caused a significant increase in ΔFENa benzamil, consistent with previous studies (Hunter et al. 2014) and suggesting upregulation of functional ENaC; the antikaliuretic effect of benzamil was similarly enhanced by sodium restriction. P2X4^−/−^ mice responded very differently: the natriuretic and antikaliuretic responses to benzamil were not enhanced by low sodium diets (Fig. 1C/D), suggesting that P2X4^−/−^ mice fail to upregulate functional ENaC activity. Plasma aldosterone concentration, which was similar in both genotypes on a standard sodium intake, was modestly increased by dietary sodium restriction (Fig. 2). This response was not different between genotypes and cannot account for the failure of P2X4^−/−^ mice to increase benzamil‐sensitive sodium reabsorption. Plasma concentration of Na^+^ and K^+^ was similar between the genotypes on a standard diet. Plasma Na^+^ was not affected in either genotype by dietary sodium restriction but this induced hypokalemia in the P2X4^−/−^ mice (Table 1).

**Figure 3:**
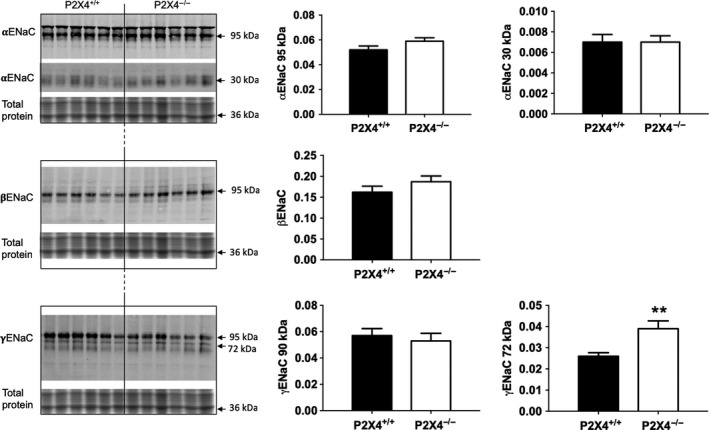
Protein abundance of ENaC subunits by Western blot analysis in P2X4^+/+^ (■) and P2X4^−/−^ (□) mice on a low Na^+^ diet for 14 days (*n* = 6 for both). Analysis of signals was performed with an infrared‐based imaging system. Data are mean ± SEM, and comparisons were made using *t*‐test. **P < 0.01.

The abundance of the ENaC subunits was measured by western blot in kidneys taken from mice fed either standard or low Na^+^ diet (Fig. 3). There was no difference between the two genotypes for any of the ENaC subunits on a standard Na^+^ diet (data not shown). On the low Na^+^ diet, no difference in the expression of *α*‐ or *β*‐ENaC subunits was found between the groups. For *γ*‐ENaC, there was no change in the expression of the uncleaved form (95 kDa). However, there was an increase in expression of the cleaved form (72 kDa) in P2X4^−/−^ mice, but not wild‐type mice.

### The effect of thiazide and bumetanide of sodium excretion

P2 receptors regulate other renal Na^+^ transporters that contribute importantly to BP regulation. We focused here on the thiazide‐sensitive NaCl cotransporter (NCC) and the bumetanide‐sensitive Na^+^, K^+^, 2Cl^‐^ co‐transporter type 2 (NKCC2), using the net natriuretic response to these diuretics as a functional readout of in vivo activity.

**Figure 4:**
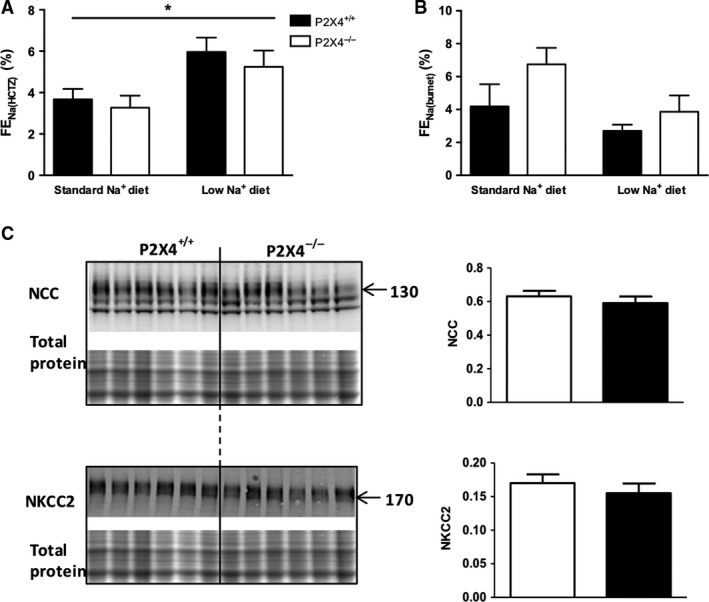
The absolute effect of (A) hydrochlorothiazide (HCTZ) and (B) bumetanide on fractional Na^+^ excretion (Δ FE_N__a(_ _HCTZ__)_ and Δ FE_N__a(bumet)_, respectively) in P2X4^+/+^ (■) and P2X4^−/−^ (□) mice on either a standard (*n* = 7 and 6 for HCTZ, 6 and 6 for bumetanide, respectively) or a low Na^+^ diet for 14 days (*n* = 7 and 6 for HCTZ, 6 and 6 for bumetanide, respectively). (C) Protein abundance of NCC and NKCC2 by Western blot analysis on a low Na^+^ diet. Analysis of signals was performed with an infrared‐based imaging system (*n* = 6 for all groups). Data are mean ± SEM, and comparisons were made with ANOVA using Bonferroni *post‐hoc* testing for (A) and (B), and *t*‐test for (C). *P < 0.05.

On a standard diet, HCTZ increased FE_Na_ to a similar extent in both genotypes. We estimate that NCC reabsorbs ~4% of the filtered sodium load, consistent with previous studies in C57BL6 mice (Hunter et al. 2014). After 2 weeks on a low sodium diet, the ΔFENa response to thiazide was enhanced, accounting now for ~6% of the filtered sodium load. This response, consistent with previous studies showing increased NCC after sodium restriction (Lai et al. 2012), was not different between genotypes (Fig. 4A).

Bumetanide induced a natriuresis in both groups of mice and overall, there was no effect of diet on delta FE_Na(bumet)_ for either genotype. There was a trend for the effect of bumetanide on delta FE_Na_ to be greater in P2X4^−/−^ mice on both diets, but this did not reach statistical significance for either Na^+^‐feeding regimen (Fig. 4B). The expression of these transporters was also assessed by Western blot analysis, and no difference was found between genotypes on either a standard (data not shown) or low Na^+^ diet (Fig. 4C).

### NOx excretion is unaltered by dietary Na^+^ restriction in P2X4^−/−^ mice

**Figure 5:**
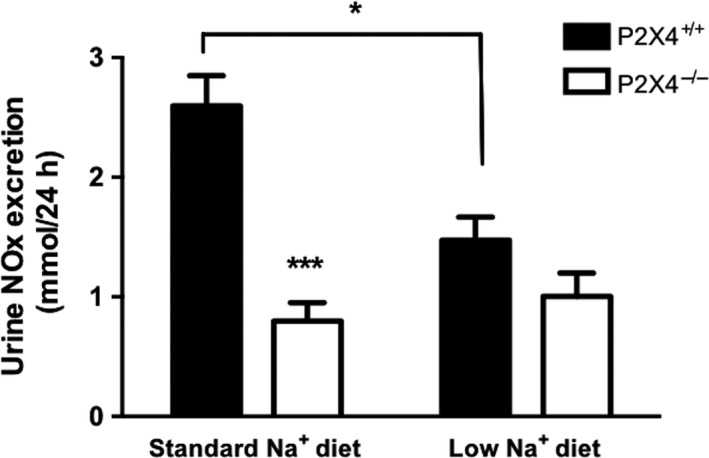
Measurements of 24 h urinary excretion of nitric oxide (NOx) in P2X4^+/+^ (■) and P2X4^−/−^ (□) mice on a standard or a low Na^+^ diet for 14 days (*n* = 6 for all groups). Data are mean ± SEM, and comparisons were made with ANOVA using Bonferroni *post‐hoc* testing. *P < 0.05; ***P < 0.001.

In our experiments on standard sodium diet, urinary NOx excretion was significantly lower in P2X4^−/−^ compared with wild‐type mice (Fig. 5). Dietary sodium restriction reduced urinary excretion of NOx in wild‐type mice, but not in P2X4^−/−^ mice (Fig. 5), suggesting that these mice do not modulate nitric oxide production with dietary salt.

## Discussion

In this study, we measured renal function and BP in P2X4^−/−^ mice under standard salt intake and after 2 weeks of adaptation to a low salt diet. The knockout mouse used here (Sim et al. 2006) was created independently from that used previously to assess vascular function (Yamamoto et al. 2006). The targeting events for these two transgenics were different: both result in loss of function of P2X4 and both mouse models are hypertensive, confirming a strong relationship between the receptor and BP. Our new findings show that high BP in P2X4^−/−^ mice is Na^+^‐sensitive, being corrected by dietary Na^+^ restriction. A limitation is that BP was measured under barbiturate anesthesia and future investigation is required to confirm the finding in conscious, unrestrained mice using radio‐telemetry.

We measured the natriuretic response to bumetanide, thiazide, and benzamil to gain insight into the in vivo activity of NKCC2, NCC, and ENaC, respectively. Surprisingly, the natriuretic response to the NKCC2 inhibitor bumetanide was similar to that of thiazide, despite the fact that ~15% of the filtered sodium load is normally reabsorbed in the thick limb of Henle. It is possible that this dose of bumetanide was not fully inhibitory; this has not been established in mice as it has for benzamil and thiazide (Hunter et al. 2014). It is also possible that the natriuretic effect is limited by delivery‐stimulated reabsorption in segments distal to the thick limb of Henle. Nevertheless, we did not see a difference in the response to bumetanide between genotypes under either diet and the response to thiazide was also no different.

In our study, we found that ENaC‐mediated sodium reabsorption accounted for ~1% of the filtered sodium load under standard sodium intake, lower than the 2‐3% reported in previous studies in C57BL6 mice (Bailey et al. 2009; Hunter et al. 2014). There were no differences between genotypes and under the standard dietary regimen, P2X4 does not influence the basal activity of ENaC, suggesting that hypertension is not directly related to enhanced ENaC function. In the original study describing the hypertensive phenotype of the P2X4^−/−^ mice, Yamamoto et al. (2006) demonstrated impaired flow‐mediated endothelial vasodilation, accompanied by reduced levels of urinary NOx. They showed that P2X4 has a role in controlling both vasodilation and vascular structural changes in response to changes in blood flow and is consistent with impaired nitric oxide production as the mechanism underlying the hypertensive phenotype. We investigated the vascular phenotype of our P2X4^−/−^ mice in vivo, confirming a significant increase in RVR and decrease in the levels of urinary NOx excretion, consistent with an underlying abnormality in vascular endothelial function.

The normalization of BP on a low Na^+^ diet indicates a degree of salt‐sensitivity in the knockout mice. The fact that the natriuretic response to benzamil did not change during sodium restriction reveals a lack of functional ENaC upregulation in these mice that was not related to a detectable change in ENaC expression. This finding is not easy to explain and does not fit with the original hypothesis that the absence of P2X4 leads to enhanced ENaC activity, thereby increasing BP. Nevertheless, the relative volume contraction observed under low dietary sodium in the knockout mice indicates a degree of sodium and water loss that might account for the reduction in BP.

What could underlie this relative decrease in ENaC function in P2X4^−/−^ mice under low Na^+^ conditions, and their seeming insensitivity to aldosterone? Low sodium increased expression of the cleaved form of *γ*‐ENaC in P2X4^−/−^ mice. Cleavage of the *α*‐ and *γ*‐ENaC subunits is associated with increased activation of the channel, particularly under physiological conditions of low Na^+^ and/or high aldosterone, and it is thought that proteolytic cleavage is important for the activation of ENaC (Frindt et al. 2001, 2008; Rossier and Stutts 2009). The *γ*‐ENaC subunit is particularly significant in proteolytic ENaC activation and it is proposed that cleavage of this subunit causes a conformational change that can induce a high open probability of the channel (Carattino et al. 2008; Diakov et al. 2008). It is possible that increased cleavage of the *γ*‐ENaC subunit under low Na^+^ conditions is a compensatory response in P2X4^−/−^ mice to normalize ENaC activity in the face of an abnormal response to aldosterone. Importantly, there is some variability for protein loading in the Western blots, which limits our ability to unequivocally interpret these data.

Both genotypes responded to dietary sodium restriction with a modest increase in aldosterone that was not statistically significant. Twenty‐four hour urinary aldosterone excretion provides a sensitive, integrated assessment of renin‐angiotensin‐aldosterone system activity but was not measured in this study. It is also possible that a more stringent restriction of dietary sodium may be required to stimulate aldosterone production. Nevertheless, we observed that only the wild‐type mice displayed the expected physiological adaption to a low salt diet and increased benzamil‐sensitive sodium reabsorption. A previous study using the Xenopus A6 principal cell‐like cell line showed that the ability of aldosterone to activate ENaC‐mediated Na^+^ reabsorption was dependent on local ATP release (Gorelik et al. 2005). The authors found that aldosterone application stimulates the release of ATP from the basolateral membrane, which in turn causes adjacent cells to contract, leading to ENaC channel opening and increased Na^+^ reabsorption. They proposed a model, whereby aldosterone‐induced ATP release stimulates basolateral P2 receptors to promote Ca^2+^ influx with ENaC activation at the apical membrane. The identity of the P2 receptor(s) involved was not addressed in this study; however, a follow‐up study using the same model with pharmacological profiling suggested involvement of P2X4 (Zhang et al. 2007), since confirmed by a similar study in A6 cells (Thai et al. 2014). These studies suggest an aldosterone‐dependent role for P2X4 in ENaC regulation. In the absence of P2X4, which can be expressed both basolaterally and apically (Wildman et al. 2008), the normal regulation of ENaC by aldosterone is impaired. This interpretation may also have some relevance to endothelial dysfunction identified in P2X4^−/−^ mice, since endothelial ENaC can influence vascular tone in a nitric oxide‐dependent manner (Kusche‐Vihrog et al. 2014).

Finally, data in rats show that nonselective blockade of P2X4/P2X7 can improve the pressure‐natriuresis response (Menzies et al. 2013), the integrated renal vascular/tubular system that regulates sodium balance, and long‐term blood pressure (Ivy and Bailey 2014). Thus, while it is likely that the raised BP in the P2X4^−/−^ mouse is primarily vascular in origin, it is clearly susceptible to normalization by dietary Na^+^ restriction, suggesting that the receptor also has a role in sodium homeostasis.

## Conflict of Interest

None.
